# Reconstruction of Sugar Metabolic Pathways of *Giardia lamblia*

**DOI:** 10.1155/2012/980829

**Published:** 2012-10-18

**Authors:** Jian Han, Lesley J. Collins

**Affiliations:** ^1^Institute of Molecular BioSciences, Massey University, Palmerston North 4442, New Zealand; ^2^Institute of Fundamental Sciences, Massey University, Palmerston North 4442, New Zealand

## Abstract

*Giardia lamblia* is an “important” pathogen of humans, but as a diplomonad excavate it is evolutionarily distant from other eukaryotes and relatively little is known about its core metabolic pathways. KEGG, the widely referenced site for providing information of metabolism, does not yet include many enzymes from *Giardia* species. Here we identify *Giardia's* core sugar metabolism using standard bioinformatic approaches. By comparing *Giardia* proteomes with known enzymes from other species, we have identified enzymes in the glycolysis pathway, as well as some enzymes involved in the TCA cycle and oxidative phosphorylation. However, the majority of enzymes from the latter two pathways were not identifiable, indicating the likely absence of these functionalities. We have also found enzymes from the *Giardia* glycolysis pathway that appear more similar to those from bacteria. Because these enzymes are different from those found in mammals, the host organisms for *Giardia*, we raise the possibility that these bacteria-like enzymes could be novel drug targets for treating *Giardia* infections.

## 1. Introduction

*Giardia lamblia* is a major cause of human waterborne diarrheal disease, infecting an estimated 10% of the world's population during their lifetime [1]. Infection is by fecal-oral transmission and is initiated by ingestion of infectious cysts in contaminated water or through person-to-person contact. After excystation, flagellated trophozoites colonize the upper small intestine where they attach to the epithelial lining but do not invade the mucosa. Around 50% of *Giardia *infections are asymptomatic, in others the major symptoms of *Giardia *infection include diarrhea, with malabsorption, dehydration, weight loss, cognitive impairment in children, and chronic fatigue in adults as well as other symptoms [2].

One of the main drugs for treating *Giardia *infection is metronidazole (Mz) [3, 4]. However, Mz treatment fails in about 20% of patients [5] and there are other issues including developing resistance from *Giardia* [2]; moreover, Mz is inactive against *Giardia *cysts [6]. The discovery and development of new therapeutics are important to expand the arsenal for controlling parasitic infection. Typically a drug target is a key molecule involved in a metabolic or signalling pathway that is specific to a disease condition or pathology, or to the infectivity or survival of a microbial pathogen [7]. Since *Giardia *is a parasite with limited metabolic diversity, a better understanding of its metabolic pathways is important to the discovery of new drug targets. Although it has been described as having some bacteria-like metabolism [6], *Giardia* displays typical eukaryotic features (e.g., cellular structure and ncRNAs such as a spliceosome [8], snoRNAs [8], and RNAi [9]). However, given the large evolutionary distance between *Giardia* and other eukaryotes, and expected genome reduction due to its parasitic lifestyle, it is no surprising that the metabolism and these eukaryotic characteristics are slightly different in *Giardia*. It is these differences that can be highly effective as drug targets.

To date, only a few metabolic pathways in *Giardia* have been described, which include some carbohydrate metabolic pathways. However, these pathways were suggested using comparative analysis and are yet to be confirmed [6, 10]. KEGGs (Kyoto Encyclopedia of Genes and Genomes) is considered one of the most important resources for understanding higher-order functional utilities of organisms from genomic information [10, 11]. However, KEGG does not yet contain many enzymes from *Giardia *[12], partly due to the incomplete annotation of *Giardia *proteins.

*Giardia *is an aerotolerant anaerobe with no mitochondria, but instead has closely related organelles called mitosomes [13, 14]. These are a reduced form of mitochondria, but whether they actually participate in ATP synthesis is currently unknown [15]. The biosynthesis of FeS clusters, which plays an important role in oxidation/reduction reactions during the electron transport chain, has been said to be the only mitochondrial function retained by mitosomes in *Giardia *[14]. Redox reactions of oxidative phosphorylation are carried out by five main protein complexes within mitochondria, named Complex I to Complex IV and ATP synthase. Some bacterial species can carry out the electron transport chain quite differently by using different electron donors, acceptors, and different enzymes. The chain may contain three proton pumps like those found in mitochondria (Complexes I, III, and IV), or it may contain only one or two pumps. Because *Giardia *is anaerobic, the majority of enzymes in the TCA cycle and mitochondrial electron transport chain are expected to be absent, but further assessments could aid in determining if there are any bacteria-like electron transport chains in *Giardia*.

In this study, a bioinformatics approach has been employed to investigate *Giardia*'s glycolysis (including gluconeogenesis and glycogen synthesis), the tricarboxylic acid cycle, (TCA cycle, also known as citric acid cycle or Krebs cycle) and oxidative phosphorylation pathways. Candidates for key enzymes in these pathways have been identified by similarity searching against all annotated enzymes from KEGG. We identified good candidates for several enzymes that were not recognized by KEGG, including phosphoglucomutase, phosphofructokinase and enzymes for ethanol fermentation. We have also determined which of the *Giardia* enzymes are more bacteria-like and which are more eukaryote-like. We use terms such as “bacteria-like” or “eukaryote-like” here to refer to enzyme amino acid sequence similarity to bacteria or eukaryotic enzymes, respectively, and not to infer any phylogenetic relationship. We have identified a number of enzymes which show differences between *Giardia* and its hosts, thus making them as potential targets for drug discovery. Our study lays the groundwork for metabolic comparisons using KEGG to enable further work towards identifying treatments for *Giardia*.

## 2. Materials and Methods

Because of the high standard of curation for genes in the KEGG database, our methodology was simple but precise. The KEGG database [12] contains networks represented by wiring diagrams of protein and other gene products responsible for various cellular processes, such as metabolism.

In KEGG, enzymes that catalyze the same reaction typically have the same enzyme commission (EC) numbers in the major databases, regardless of their homology. Enzymes with the same EC number may show significant sequence and structural similarity. However, in some cases enzymes with the same activity (i.e., same EC number) can be associated with different phylogenetic lineages and have different catalytic mechanisms with little structural similarity. We used EC numbers during this study because enzymes can have variation on their names, but the EC numbers will remain the same (e.g., “phosphohexose isomerase” can also be referred to as “phosphoglucose isomerase” or “Glucose-6-phosphate isomerase,” but the EC number will always be “EC 5.3.1.9”). 

The “genes.pep” file was downloaded from KEGG (accessed January 2011) [12], containing all the sequences in the KEGG database in FASTA format (a total of 5,338,631 sequences, with the EC number included in the annotation of each protein). This file made it possible to pull out all sequences belonging to the same EC number from different organisms. For each EC group, enzymes from all species were collated into a single FASTA file by the use of a Perl script, the *Giardia *protein database was then BLAST searched (program “blastp” default parameters used) [16] against this FASTA file, and the proteins with the highest bit-score were recorded. A metabolic map of the pathway was generated according to bit-scores (the log scaled score given to alignments by BLAST, higher numbers correspond to higher similarity) of the hits for each enzyme. Hits with bit-scores higher than 300 were considered to be high-quality candidates for the enzyme, and hits with scores between 100 and 300 were considered as lower quality candidates. This procedure was repeated for each enzyme in the glycolysis, TCA cycle, and oxidative phosphorylation pathways. EC numbers for all enzymes detected in *Giardia *in these pathways are given in Tables S1–S3 available online at doi: 10.155/2012/980829.

## 3. Results

### 3.1. Glycolysis and Gluconeogenesis

**Figure 1:**
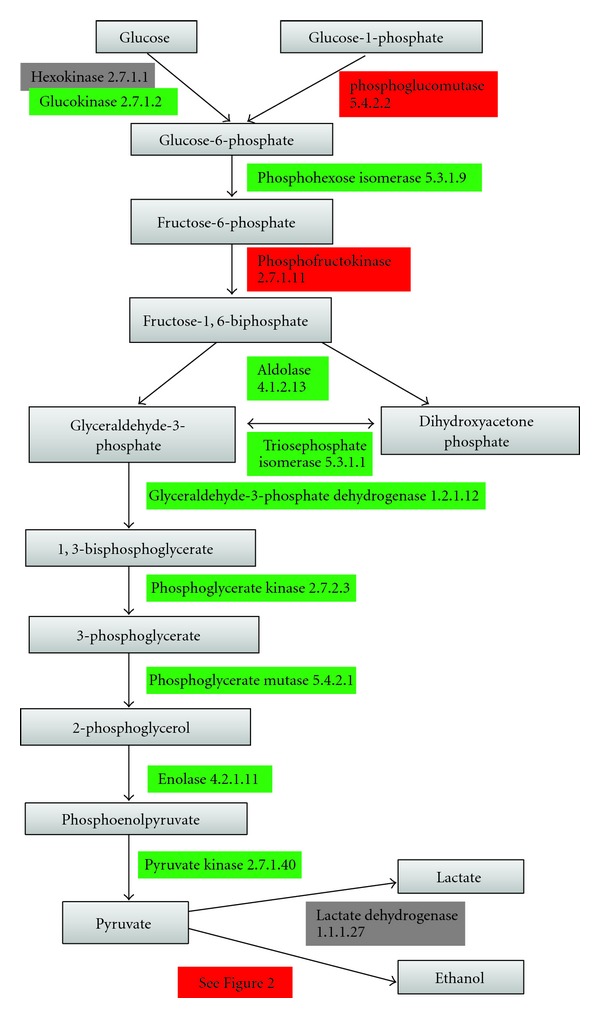
Glycolysis in *Giardia*. The diagram indicated which enzymes have been directly identified by KEGG (green), which have been identified during this study (red), and which are not present (grey). As can be seen, most glycolytic enzymes are present in *Giardia*. A more technical representation of this image is present in Figure S1. Key. The metabolites are labeled in grey boxes, the enzymes which catalyze reactions from one metabolite to another are shown in rectangles with their EC number indicated, and are colored according to their similarity to enzymes of other species: green: enzymatic function registered in KEGG; red: found in *Giardia* with bit-score >300 and these are enzyme candidates with fairly high degrees of certainty; grey: found in *Giardia* with bit-score <100; there were no enzymes found in *Giardia *with bit-scores between 100–300 in this pathway.

As expected, because glycolytic proteins are highly conserved in eukaryotes, the major enzymes in the backbone of the glycolysis pathway were found in the *Giardia *genome (see Figure 1). However, an unexpected feature is that most of these enzymes showed greater similarity to their bacterial orthologues than their eukaryotic orthologues (refer to supplementary data); we found that some of these bacteria-like enzymes (e.g., phosphofructokinase, EC 2.7.1.11) are also often present in other eukaryotic protists (*Toxoplasma*, *Tetrahymena*,* Trypanosoma*, *Plasmodium* and *Trichomonas*). There were a few eukaryote-like enzymes from the glycolysis pathway detected, including phosphoglucomutase (EC 5.4.2.2), phosphoglycerate kinase (EC 2.7.2.3), dihydrolipoyllysine-residue acetyltransferase (EC 2.3.1.12), and enolase (EC 4.2.1.11). These results are explained in more detail below.

Phosphoglucomutase (PGM, EC 5.4.2.2) facilitates the interconversion of glucose-1-phosphate and glucose-6-phosphate. *Giardia* protein GL50803_17254 showed high similarity to the PGM from eukaryotes (bit-score of 310). Experimental evidence from Mitra et al. [17] indicates that this protein has phosphoglucomutase activity, validating in part the potential of our method for finding new enzymes. Another glycolytic enzyme not yet in KEGG to *Giardia*, phosphofructokinase was also recovered, with the *Giardia* protein GL50803_14993 showing strong similarity to phosphofructokinases from many bacterial species. KEGGs have assigned the EC number “EC 2.7.1.90” to this protein, suggesting that this is a pyrophosphate based-phosphofructokinase.

Two enzymes are responsible for the interconversion of glucose to glucose-6-phosphate, namely, hexokinase (EC 2.7.1.1) and its isozyme glucokinase (EC 2.7.1.2). The difference is that glucokinase has a lower affinity for glucose than hexokinase. A *Giardia *glucokinase has been described by KEGG (GL50803_8826), with this protein showing more similarity (i.e., higher bit-score) to the bacterial (cyanobacteria) glucokinase than eukaryotic glucokinase. *Tetrahymena thermophila* (a free living protozoan species) also has a similar type of glucokinase. As yet we do not know why *Giardia *has a lower affinity enzyme, but one possible reason is that the trophozoite living environment (intestines of animals) provides a generous glucose supply so a high-affinity enzyme is not required. We did not identify any hexokinase from *Giardia* although one fungal protein (uma:UM03093.1, “uma” indicates the species: *Ustilago maydis* and “UM03093.1” is the accession number of the protein) was mislabeled as a hexokinase by KEGGs, and returned a *Giardia *protein with high similarity. Upon further analysis of uma:UM03093.1 by using genomic resource databases [18] and similarity search, we determined that the fungal and *Giardia* proteins were in fact false positives for hexokinase. In light of this we suggest that all conversion of glucose to glucose-6P is carried out by glucokinase exclusively in *Giardia*.

The conversion of pyruvate to lactate is catalyzed by lactate dehydrogenase (EC 1.1.1.27), and the coupled reaction also oxidizes coenzyme NADH to NAD^+^. This reaction occurs in lactate fermenting bacteria and in eukaryotes such as humans in the absence of oxygen, to provide a constant supply of oxidized form of coenzyme NAD^+^ for glycolysis. The only *Giardia *protein with high bit-score to any known lactate dehydrogenase is GL50803_17325 with a bit-score of 161 against one (and only one) lactate dehydrogenase from *Toxoplasma gondii* (also a parasitic protozoan). However, further analysis indicated that tgo:TGME49_060600 may have been incorrectly assigned by KEGG (in the same manner as described above for hexokinase). Our results here suggest that *Giardia *lacks lactate dehydrogenase and that lactic acid fermentation does not take place in *Giardia*. Instead the reoxidation of coenzyme NADH to NAD^+^ is performed by ethanol fermentation (discussed later).

Pyruvate synthase (EC 1.2.7.1) is also known as pyruvate:ferredoxin oxidoreductase (PFOR). This is an alternative enzyme to the pyruvate dehydrogenase complex (formed together by EC 1.2.4.1, EC 2.3.1.12, and EC 1.8.1.4) found in mammals. PFOR is able to oxidise pyruvate to acetyl-CoA, but utilizes ferredoxin rather than NAD^+^ as the electron acceptor. The PFOR of *Giardia *is GL50803_17063 and is the main target for the drug Metronidazole (Mz) [4]. The selective toxicity of Mz is achieved because only the parasite has PFOR.

**Figure 2:**
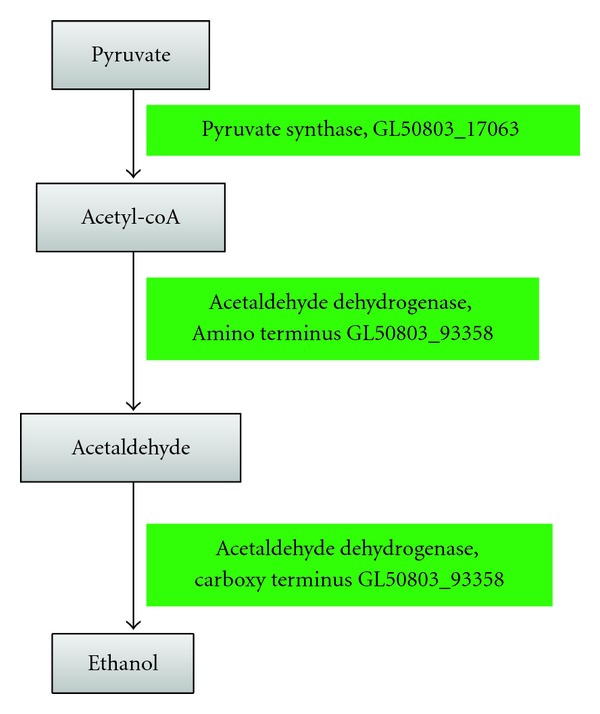
A possible ethanol fermenting pathway of *Giardia. *Pyruvate is metabolized into acetyl-CoA, and acetyl-CoA is subsequently converted to acetaldehyde and alcohol by a bifunctional acetaldehyde-CoA and alcohol dehydrogenase.

*Giardia *performs ethanol fermentation to maintain a constant supply of NAD^+^, but this pathway is different from that found in some bacteria and yeast in that it converts pyruvate into acetaldehyde and then into ethanol. *Giardia *seems to be unable to convert pyruvate directly to acetaldehyde because pyruvate decarboxylase (EC 4.1.1.1) is noticeably absent. *Giardia *performs ethanol fermentation by first converting pyruvate to acetyl-CoA (by pyruvate synthase, EC 1.2.7.1), then to acetaldehyde and finally to ethanol (see Figure 2). It has been reported [19, 20] that a *Giardia* enzyme has acetaldehyde dehydrogenase (EC 1.2.1.10) activity in the aminoterminus which catalyzes the conversion of acetyl-CoA to acetaldehyde, and alcohol dehydrogenase (EC 1.1.1.1) activity in the carboxy-terminus which catalyses the conversion of acetaldehyde to ethanol, but the paper did not include the accession number used to identify the protein. We identified the aforementioned protein, as GL50803_93358 which scored high bit-scores (870) for both alcohol dehydrogenase and acetaldehyde dehydrogenase. The best-scored hit of this protein, tel:tlr0227, is also incidentally a bifunctional acetaldehyde-CoA and alcohol dehydrogenase from the cyanobacterium* Thermosynechococcus elongates*.

We also found acetyl-CoA synthetase (EC 6.2.1.13) in *Giardia *(GL50803_13608), indicating that pyruvate can also be converted to acetyl-CoA and then to acetate. Experimental evidence suggests that the metabolism of trophozoites is markedly affected by small changes in oxygen concentration [21]. Under anaerobic conditions, ethanol is the major product of carbohydrate metabolism and under aerobic conditions, alanine and acetate are the predominant products of energy metabolism. Thus, the pyruvate metabolism pathway appears to be flexible for dealing with different aerobic/anaerobic environments [21].

We have identified enzymes which are significantly different from that of the host, as large amount enzymes in the glycolytic pathway are more closely related to that of archaea and bacteria, and thus different from those of eukaryotes. The *Giardia* enzymes that are different from eukaryotic enzymes and hence are possibilities for future drug discovery are listed in Table 2.

There is no recent literature on the presence of the gluconeogenesis pathway in *Giardia*. It has been suggested that gluconeogenesis may occur during encystation, when glucose uptake decreases substantially and *Giardia *gains its energy by uptaking amino acids (aspartate) followed by gluconeogenesis [6]. The gluconeogenesis pathway shares a number of identical enzymes with the glycolysis pathway. There are three subtle differences: first the reaction catalysed by pyruvate kinase (converting phosphoenolpyruvate to pyruvate) is irreversible, but pyruvate carboxylase (EC 6.4.1.1) and phosphoenolpyruvate carboxykinase (PEPCK, EC 4.1.1.32) can convert pyruvate into oxaloacetate and then back to phosphoenolpyruvate, which can be used for gluconeogenesis. Proteins with similarities to both of these enzymes were found in *Giardia *in this analysis, although pyruvate carboxylase has not yet been described in *Giardia* by KEGG. The second enzyme where gluconeogenesis differs from glycolysis is fructose bisphosphatase (EC 3.1.3.11) which converts fructose-1,6-bisphosphate to fructose-6-phosphate in gluconeogenesis (the reverse of the reaction catalysed by phosphofructokinase in glycolysis). In Giardia, only the protein GL50803_17316 has some low similarity (bit-score of 99) to fructose bisphosphatase. Given this low similarity, it is likely this protein does not have fructose bisphosphatase enzymatic activity. Lastly, in the reverse of the reaction that is catalysed by glucokinase, no candidates for glucose-6-phosphatase (EC 3.1.3.9) were recovered. Overall, our analysis suggests that two key gluconeogenic enzymes are absent and that Giardia does not have the entire set of enzymes required to perform gluconeogenesis. Giardia may take up amino acids for energy, but may not convert it all the way back to glucose, and instead the amino acids are possibly converted to pyruvate or oxaloacetate to obtain limited energy (via the likes of the arginine dihydrolase pathway).

Although unable to regenerate glucose, as we looked for required enzymes to synthesize glycogen from glucose (glycogenesis), *Giardia* does appear to have all of them present: UTP-glucose-1-phosphate uridylyltransferase (EC 2.7.7.9) and glycogen synthase (EC 2.4.1.11) have both been noted by KEGG. Combining this result with previous reports that glycogen has been found to be present in trophozoites [22], we suggest that *Giardia* is able to generate glycogen from glucose to serve as an energy reserve.

In summary, *Giardia* is able to perform glycolysis, using glycolytic catabolic reactions to provide energy for the organism. *Giardia* is also able to synthesis glycogen from glucose to create an energy reserve. However, it appears that *Giardia* is unable to perform gluconeogenesis to generate glucose from pyruvate.

### 3.2. Tricarboxylic Acid Cycle

**Figure 3:**
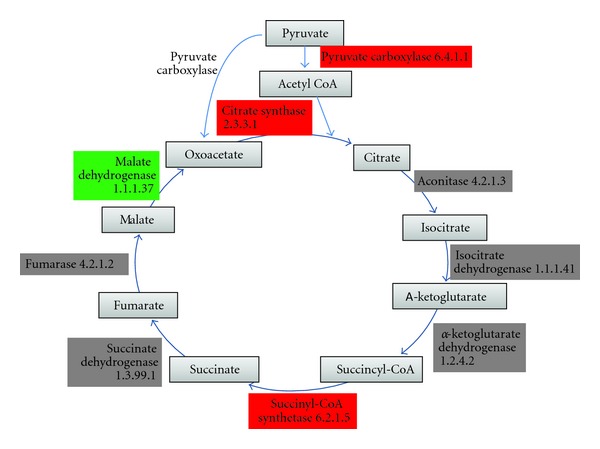
TCA cycle enzymes in *Giardia.* Limited candidates were found for enzymes in the TCA cycle. A more technical representation of this image is present in Figure S2. Key as for Figure 1.

Most of the enzymes in the TCA cycle were not detected in *Giardia *(Figure 3). This was expected because *Giardia *is an anaerobe and lacks the mitochondria in which the TCA cycle typically operates in other eukaryotes. Those enzymes that are present in *Giardia *are also part of alternative metabolic pathways (pyruvate synthase (EC 1.2.7.1), pyruvate carboxylase (EC 6.4.1.1), and PEPCK (EC 4.1.1.32) are all in the glycolysis pathway). The presence of citrate synthase (EC 2.3.3.1) and malate dehydrogenase (EC 1.1.1.37) is expected because citrate and malate are important intermediates involved in the metabolism of highly interconnected cellular metabolites. Thus it is possible that *Giardia *will need pyruvate synthase, pyruvate carboxylase, and PEPCK to metabolise malate and citrate.

The best candidate for succinyl-CoA synthetase (EC 6.2.1.5) is GL50803_13608, which also has similarity to acetyl-CoA synthetase (EC 6.2.1.13). The substrates for both enzymes are similar, and it is more likely that this protein is an acetyl-CoA synthetase because of the higher bit-score (507 for acetyl-CoA synthetase versus 435 for succinyl-CoA synthetase) and the fact that GiardiaDB labelled this as acetyl-CoA synthetase. Overall, the lack of the majority of the enzymes in the pathway suggests the absence of the TCA cycle in *Giardia*.

### 3.3. Oxidative Phosphorylation

**Figure 4:**
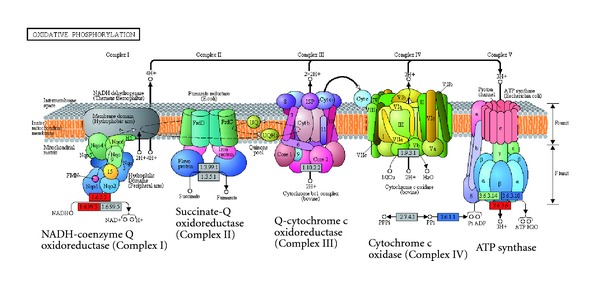
The Oxidative phosphorylation pathway in *Giardia*. Only proteins with similarity to enzymes in Complex I and Complex V (ATP synthase) were found in *Giardia*. Key as for Figure 1, except that the blue enzyme boxes indicate the enzyme has registered in KEGG but was shown in their map of *Giardia* metabolic pathways. The template image was downloaded from KEGG.

This pathway is harder than the other pathways to analyze, because many enzyme subunits with the same EC group form the multimeric complexes involved in the pathway (e.g., Complex I contains as many as 45 peptides in metazoans). We do not expect *Giardia *to have a typical electron transport chain because they lack mitochondria, and there is likely to be a limited supply of reduced NADH due to the absence of the TCA cycle. The main components of a typical oxidative phosphorylation pathway are shown in Figure 4.

Our analysis showed that Complex II (succinate dehydrogenase), Complex III (ubiquinol-cytochrome-c reductase), and Complex IV (cytochrome c oxidase) are clearly absent in *Giardia*. Proteins with similarity to Complex I (NADH dehydrogenase, EC 1.6.5.3, and EC 1.6.99.3, the difference between the two EC groups is that the former uses ubiquinone as the electron acceptor and the later does not have a specified electron acceptor) were recovered from *Giardia* (e.g., GL50803_6304 and GL50803_33769), but given that this complex contains up to 45 peptides, and very few similar proteins have been found in *Giardia*, it is unlikely that *Giardia* has the entire Complex I.

The ATP synthase (EC 3.6.3.34, labelled as Complex V in Figure 4) was determined to be present. As many as 14 proteins (GL50803_10530, GL50803_12216, GL50803_13000, GL50803_13603, GL50803_14660, GL50803_14961, GL50803_15598, GL50803_18470, GL50803_30851, GL50803_3678, GL50803_7532, GL50803_8367, GL50803_8559, GL50803_87058) have been assigned to this EC group. These 14 proteins make up the vacuolar (V-type) ATPase [23]. The V-type is different from F_1_F_O_ (F-type) ATPase, which is present in the plasma membrane of bacteria, the inner membrane of mitochondria, and the thylakoid membranes of chloroplasts. The V-ATPase is present in the endomembrane systems of eukaryotes: vacuoles, Golgi apparatus, and coated vesicles. V-type ATPases build up a H^+^ gradient across the membrane via ATP hydrolysis to transport solutes, or to lower the pH inside the endomembrane system, in reverse of reactions catalysed by F-ATPase [23]. Its function is to generate a proton gradient rather than utilizing the proton gradient to harvest ATP. The other two ATP synthase enzymes present in *Giardia *were the H^+^/K^+^-exchanging ATPase (EC 3.6.3.10) and H^+^-exporting ATPase (EC 3.5.3.6). These two enzymes function as transporters rather than ATP generators. No F-type ATPases were recovered from *Giardia *during our study, indicating a possibility that there is no ATP-producing ATP synthase. Overall the lack of Complexes I, II, III, and IV suggests that *Giardia* is unable to actively generate the proton gradient that is vital for generation of ATPs by F-ATP synthase.

Some bacterial species can carry out the electron transport chain differently (i.e., during anaerobic respiration), by using different electron donors and acceptors and therefore different enzymes (e.g., *Escherichia coli* can use a large number of electron donor/acceptor pairs such as fumarate/succinate, or pyruvate/lactate [24]). We cannot rule out that *Giardia* might have such a mechanism or an as yet completely novel mechanism, but an F-type ATP synthase is still lacking, suggesting that *Giardia* is unable to mass produce ATP by using a the typical eukaryotic electron transport chain pathway.

## 4. Discussion

Overall, we have been able to reconstruct a number of sugar-related metabolic pathways for *Giardia lamblia* and highlight notable enzyme absences from these pathways. The glycolytic enzymes from *Giardia* bear a stronger similarity with bacterial enzymes, rather than with eukaryotic or archaeal enzymes (except for phosphoglucomutase and phosphoglycerate kinase which are more similar to those found in eukaryotes). Only a few enzymes were identified from the TCA cycle and oxidative phosphorylation, indicating the likely absence of these pathways. 

This approach of analysing metabolic pathways could, in theory, be applied to any organism with genome information but limited annotation. The advantage of using this approach is that it is reasonably quick to give an indication of which pathways are likely to be present and which ones are not. There are however some limitations: for a few proteins, KEGG can allocate wrong EC numbers which will result in false positives if users are not familiar with the pathways. False positives can also occur if one EC group is very similar to another EC group (such as in the case of succinyl-CoA synthetase and acetyl-CoA synthetase). KEGG is a database that is still growing and as yet does not have the enzymes from all species. It is expected that enzyme candidates may not be recovered if they are from a species extremely different from the known enzymes and species. We expect this issue will decrease with time as enzymatic studies on species such as *Giardia* add to the improvement of KEGG annotations.

The overall picture of *Giardia *indicates that glucose is absorbed from the host and metabolised into pyruvate through glycolysis and after that, in order to regenerate the oxidised form of coenzyme NAD^+^, pyruvate is reduced to ethanol, alanine, or acetate depending on the availability of oxygen. Under aerobic conditions, pyruvate is converted to alanine by a transamination reaction, or to acetate by acetyl-CoA synthetase. Also under anaerobic conditions, pyruvate is metabolised to acetyl-CoA by PFOR, and subsequently into acetaldehyde and ethanol. The TCA cycle and oxidative phosphorylation do not appear to occur. These latter results were not completely unexpected since we already know that *Giardia *has an anaerobic life style and has undergone genome reduction (i.e., a smaller genome with fewer unnecessary enzymes will give the parasite advantage when replicating) [10].

*Giardia *shares many metabolic attributes of bacteria, including its fermentative energy metabolism which relies heavily on pyrophosphate rather than adenosine triphosphate. Morrison et al. (2007) looked into *Giardia*'s metabolic repertoire briefly when the *Giardia *genome project was completed. Their results indicated that *Giardia*'s sugar metabolic pathways contained a mixture of eukaryote-like (enzymes that appeared more similar in sequence to those enzymes found in eukaryotes) and bacteria-like enzymes [10]. Morrison et al. (2007) indicated that about half of glycolytic enzymes are eukaryote-like [10], but they did not distinguish between typical eukaryotic enzymes (i.e., those well studied in mammals, yeasts, and plants) and enzymes from eukaryotic protists. Our study has considered protists separately from other eukaryotes, because frequently these eukaryotic protists have prokaryote-like enzymes rather than those from typically studied eukaryotes. Some reasons for *Giardia* having a sizable number of bacteria-like enzymes include the possibilities that mitochondria genes migrated to the nucleus with the loss of this organelle [25] lateral gene transfer of bacterial genes [26] or that the eukaryotic set of enzymes arose after their divergence from the ancestral eukaryote. There are still many evolutionary questions surrounding *Giardia* and it is expected that the clarification of its somewhat “atypical” metabolism will aid this research.

The glycolysis pathway occurs, in nearly all organisms with minor variations [27]. So we ask very briefly if the enzymes in the glycolysis pathway are also conserved in all organisms. We have compared the *Giardia* annotated proteins (4889 in total) against 28 bacterial, 12 archaeal species, and 17 other eukaryotic species, and identified four groups of proteins according to the conservation of the proteins in the three domains: Group A contains 37 *Giardia *proteins that are conserved in all three domains of life; Group B contains 849 *Giardia *proteins that are found in all eukaryotes; Group C contains 274 eukaryotic signature proteins [28, 29], which are proteins conserved in all eukaryotes, but not found in any archaea or bacteria; and finally Group D contains 278 *Escherichia coli* proteins conserved in all bacteria species.

The candidates of glycolytic enzymes (20 in total) were compared with the above four groups of proteins. None of the glycolytic enzymes matched were matched to Group A (conserved in all three domains), Group C (eukaryotic signature proteins), or Group D (conserved in all bacterial species). However, there were six candidates matched to Group B (conserved in all eukaryotic species, Table 1).

These results could be explained because *Giardia *contains a mixture of eukaryote-like and prokaryotic-like enzymes in glycolysis and that glycolysis in bacteria occurs in diverse forms. This means that none of the *Giardia*'s bacteria-like glycolytic enzymes are likely to be universal to all bacteria and thus less likely to be found matched to those in Group A or Group D. The eukaryotic glycolytic enzymes are more conserved across eukaryotes, and thus some of *Giardia*'s eukaryote-like glycolytic enzymes were found to be conserved in all eukaryotes. However, homologues of these enzymes conserved in all eukaryotes are also found in some branches of bacteria, hence they did not show up in Group C (eukaryotic signature proteins). This result is due to the large variety of glycolytic enzymes present in bacteria. This above work is merely indicative at this stage and will be the basis for future study.

More pathways, such as those involved in amino acid metabolism, and the RNA degradation pathway can be analysed using this method, adding more pieces to the puzzle of *Giardia*'s metabolism. This study also identified *Giardia *candidates for enzymes that had not been recognized before. They bear high similarity to known enzymes of their classes, and although the actual functions of these enzymes have not been confirmed, our work gives direction to future experimental confirmation with activity assays. 

Typically a drug target is a key molecule for the infectivity or survival of a microbial pathogen. Selective toxicity would be best achieved if the parasite has a key enzyme that humans do not have or which is remarkably different from the host. For example, PFOR is found in *Giardia*, but the host (human or mammal) uses the pyruvate dehydrogenase complex to perform the same reaction, and thus drugs targeting PFOR such as metronidazole have been designed. From the glycolytic pathway, we have identified enzymes which are significantly different in *Giardia* from those in the host (see Table 2), including glucokinase and phosphofructokinase. Glucokinase has been investigated as a drug target for type 2 diabetes [30], and its potential to be a target for parasite infection is as yet uncertain. Phosphofructokinase has been suggested as a drug target for *Entamoeba histolytica* by Byington et al. [31], and they designed a competitive inhibitor of phosphofructokinase, with the drug inhibiting the growth of the parasite* in vitro*. These enzymes, and especially those that can be compensated in the host by alternative pathways, hold the possibility of new targets for drugs effective against *Giardia*. An even better understanding of this parasite's metabolism will surely provide more ammunition against this worldwide parasitic problem.

## Supplementary Material

Supplementary material. KEGG diagrams of Giardia glycolysis and citric acid cycle. We have indicated which enzymes have already been identified by KEGG and which ones we have discovered new candidates for and to which degree of confidence.

## Figures and Tables

**Table 1:** *Giardia *glycolytic enzyme candidates maintained in all eukaryotes.

Protein	Enzyme name	EC number
GL50803_11118	Enolase	4.2.1.11
GL50803_7260	Alcohol dehydrogenase	1.1.1.2
GL50803_7982	Aldose 1-epimerase	5.1.3.3
GL50803_90872	Phosphoglycerate kinase	2.7.2.3
GL50803_9115	Glucose-6-phosphate isomerase	5.3.1.9
GL50803_93938	Triosephosphate isomerase	5.3.1.1

**Table 2:** Bacteria-like *Giardia *enzymes in glycolysis pathway.

EC	Enzyme name	Best candidate	Bit score	*E* value
2.7.1.2^‡^	Glucokinase	GL50803_8826	393	2.00 × 10^−110^
2.7.1.11	Phosphofructokinase	GL50803_14993	429	9.00 × 10^−122^
4.1.2.13^‡^	Aldolase	GL50803_11043	390	3.00 × 10^−110^
1.2.1.59	Glyceraldehyde-3-phosphate dehydrogenase (NAD(P)^+^)	GL50803_6687	326	6.00 × 10^−92^
1.2.7.6	Glyceraldehyde-3-phosphate dehydrogenase	GL50803_6687	315	5.00 × 10^−89^
5.4.2.1^†^	Phosphoglycerate mutase	GL50803_8822	551	4.00 × 10^−142^
1.2.7.1	Pyruvate synthase	GL50803_17063	1008	0.0
6.2.1.13	Acetyl-CoA synthetase (ADP-forming)	GL50803_13608	507	5.00 × 10^−146^
1.1.1.1	Alcohol dehydrogenase	GL50803_93358	870	0.0
eutG	Ethanol : NAD^+^ oxidoreductase	GL50803_93358	717	0.0
1.2.1.10	Acetaldehyde dehydrogenase	GL50803_93358	870	0.0
