# Supplementary material for: Reconstruction of Sugar Metabolic Pathways of Giardia lamblia
Source: Int J Proteomics. 2012 Oct 18;2012:980829. doi: 10.1155/2012/980829 (PMC3483818; doi:10.1155/2012/980829)
Supplement: Supplementary file 1 — Supplementary material. KEGG diagrams of Giardia glycolysis and citric acid cycle. We have indicated which enzymes have already been identified by KEGG and which ones we have discovered new candidates for and to which degree of confidence. [file 980829.f1.docx]

**Figure S1**

**
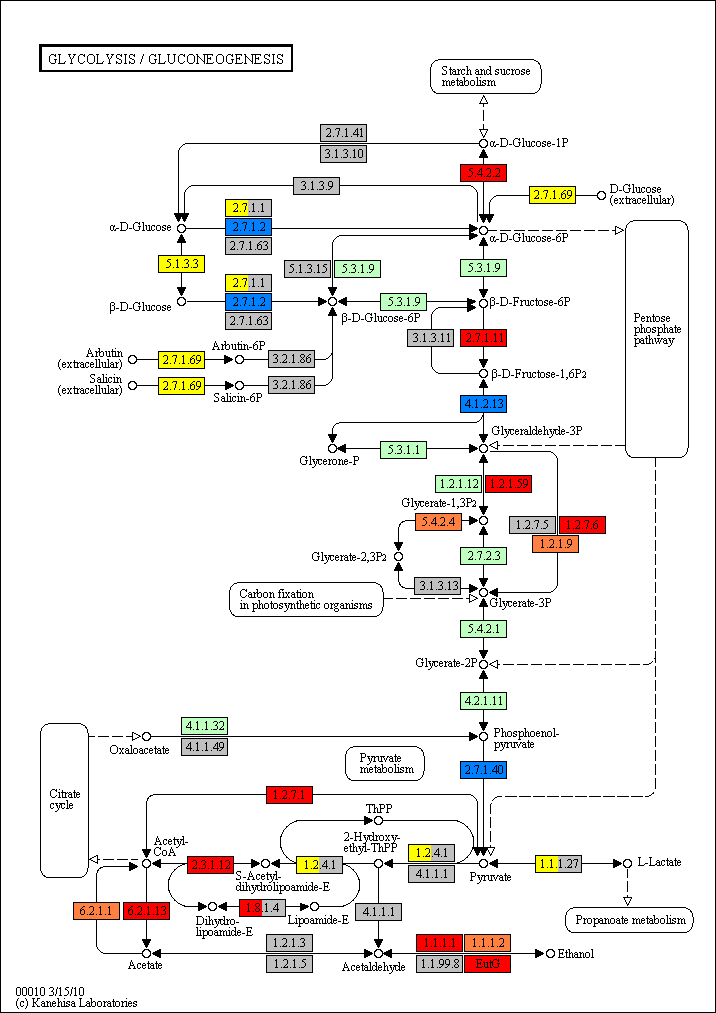
**

**Figure S2**

**
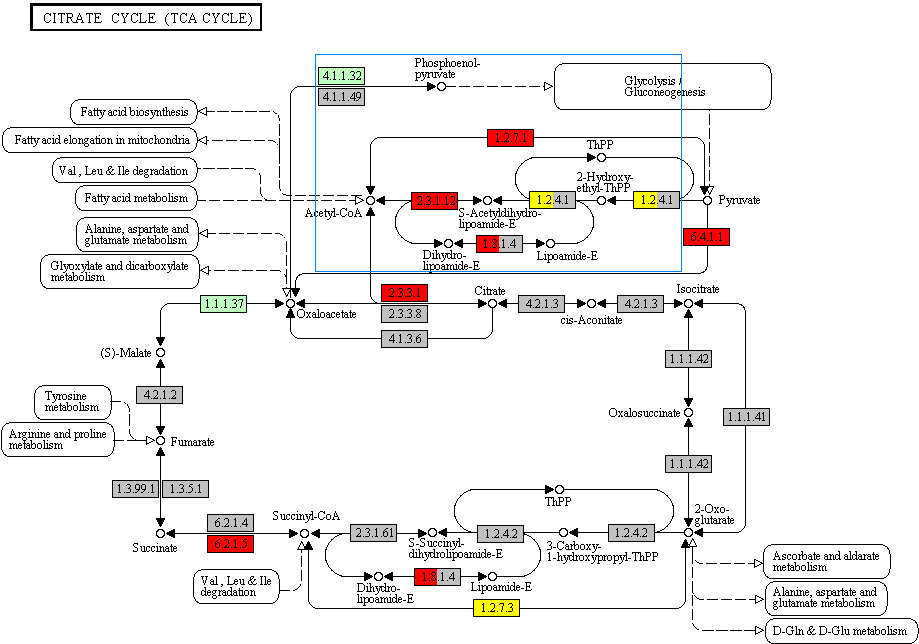
**

**Supplementary figures**

##### **Figure S1** KEGG diagram of glycolytic enzymes in *Giardia* indicating which enzymes have been directly identified by KEGG (green and blue), and which have been identified during this study (red, orange and yellow). Key: The metabolites are labelled and shown as small circles, the enzyme which catalyse reactions from one metabolite to another are shown in rectangles, with their EC number indicated. The *Giardia* enzymes are coloured according to their similarity to enzymes of other species: green: enzymatic function registered in KEGG; blue: EC number registered in KEGG but was in the KEGG Giardia glycolysis pathway (This is because KEGG also have a KEGG orthology (KO) number, which indicate if there are different groups of enzymes with same EC number, some lesser studied enzymes such as those of *Giardia*’s have not been given a KO number, and KEGG was conservative not to show them on the map of *Giardia* metabolic pathway. In this case the enzymes were coloured blue in our figures); red: found in *Giardia* with score >300, these are enzyme candidates with fairly high degrees of certainty; orange: found in *Giardia* with score 200-300; yellow: found in *Giardia* with score 100-200, grey: found in *Giardia* with score <100. Half painted grey indicates the result is very likely a false positive. The template image was downloaded from KEGG.

**Figure S2** TCA cycle enzymes in *Giardia.* Colouring key is same as Figure S1. The bright blue box indicates this part of the pathway is also part of the glycolysis pathway in Figure S1. The template image was downloaded from KEGG.
