# Supplementary material for: Reconstruction of Sugar Metabolic Pathways of Giardia lamblia
Source: Int J Proteomics. 2012 Oct 18;2012:980829. doi: 10.1155/2012/980829 (PMC3483818; doi:10.1155/2012/980829)
Supplement: Supplementary file 2 [file 980829.f2.docx]

## Supplementary data

### 1. Enzymes from the glycolysis pathway in *Giardia*

The enzymes are ordered in the approximately direction of the metabolic flux. † indicates this enzyme is already in KEGG *Giardia* glycolysis pathway, ‡ indicates the *Giardia* enzyme has already giving the EC number, but was not in the KEGG *Giardia* glycolysis pathway for unknown reason, ! indicates high possibility of false positive result. The last column indicates whether the *Giardia* enzyme is more homologous to bacterial (B), archaeal (A) or eukaryotic (E) enzymes, P indicates eukaryotic protists, which are considered separately from other eukaryotes.

| EC | Enzyme name | Sequences in KEGG (#) | Best candidate | Bit-score | Evalue | Domains |
| --- | --- | --- | --- | --- | --- | --- |
| 2.7.1.41 | glucose-1-phosphate phosphodismutase | 11 | GL50803_14038 | 60.1 | 6.00E-13 | - |
| 3.1.3.10 | glucose-1-phosphatase | 84 | GL50803_7556 | 31.6 | 0.003 | - |
| 5.4.2.2 | phosphoglucomutase | 929 | GL50803_17254 | 310 | 9.00E-86 | E, P |
| 3.1.3.9 | glucose-6-phosphatase | 28 | GL50803_5631 | 30.8 | 0.004 | - |
| 2.7.1.1! | hexokinase | 305 | GL50803_7260 | 179 | 5.00E-47 | - |
| 2.7.1.2‡ | glucokinase | 1110 | GL50803_8826 | 393 | 2.00E-110 | B, P |
| 5.1.3.3 | aldose 1-epimerase | 736 | GL50803_7982 | 124 | 3.00E-30 | B, E |
| 5.1.3.15 | glucose-6-phosphate 1-epimerase | 6 | GL50803_9115 | 93.2 | 2.00E-22 | - |
| 5.3.1.9† | glucose-6-phosphate isomerase | 1261 | GL50803_9115 | 394 | 5.00E-111 | P, B, E |
| 2.7.1.69 | glucose permease | 8977 | GL50803_9909 | 130 | 6.00E-31 | B |
| 3.2.1.86 | 6-phospho-beta-glucosidase | 788 | GL50803_35487 | 38.9 | 0.004 | - |
| 3.1.3.11 | fructose-bisphosphatase | 1286 | GL50803_17316 | 99 | 3.00E-22 | B |
| 2.7.1.11 | phosphofructokinase | 1172 | GL50803_14993 | 429 | 9.00E-122 | P, B |
| 4.1.2.13‡ | aldolase | 1839 | GL50803_11043 | 390 | 3.00E-110 | B, P |
| 5.3.1.1† | triosephosphate isomerase | 1352 | GL50803_93938 | 348 | 8.00E-98 | B, P, E |
| 1.2.1.12† | glyceraldehyde-3-phosphate dehydrogenase | 1872 | GL50803_17043 | 270 | 9.00E-74 | B, E, P |
| 1.2.1.12† | glyceraldehyde-3-phosphate dehydrogenase | 1872 | GL50803_6687 | 459 | 5.00E-131 | E, B, P |
| 1.2.1.59 | glyceraldehyde-3-phosphate dehydrogenase (NAD(P)^+^) | 138 | GL50803_6687 | 326 | 6.00E-92 | B |
| 5.4.2.4 | bisphosphoglycerate mutase | 60 | GL50803_8822 | 278 | 1.00E-77 | B, E |
| 2.7.2.3† | phosphoglycerate kinase | 1289 | GL50803_90872 | 453 | 4.00E-129 | E |
| 3.1.3.13 | bisphosphoglycerate phosphatase | 31 | GL50803_135885 | 45.8 | 2.00E-08 | - |
| 1.2.7.5 | aldehyde ferredoxin oxidoreductase | 330 | GL50803_13616 | 47.4 | 4.00E-07 | - |
| 1.2.7.6 | glyceraldehyde-3-phosphate dehydrogenase | 26 | GL50803_6687 | 315 | 5.00E-89 | B |
| 1.2.1.9 | glyceraldehyde-3-phosphate dehydrogenase (NADP^+^) | 168 | GL50803_6687 | 209 | 2.00E-56 | B |
| 5.4.2.1† | phosphoglycerate mutase | 2987 | GL50803_8822 | 551 | 4.00E-142 | B |
| 4.2.1.11† | enolase | 1329 | GL50803_11118 | 455 | 1.00E-129 | P, E |
| 4.1.1.32† | phosphoenolpyruvate carboxykinase (GTP) | 322 | GL50803_10623 | 470 | 2.00E-134 | A, E, B |
| 4.1.1.49 | phosphoenolpyruvate carboxykinase (ATP) | 554 | GL50803_10623 | 41.6 | 4.00E-05 | - |
| 2.7.1.40‡ | pyruvate kinase | 1575 | GL50803_3206 | 1243 | 0 |  |
| 1.1.1.27! | L-lactate dehydrogenase | 658 | GL50803_17325 | 161 | 3.00E-41 | - |
| 1.2.7.1 | pyruvate synthase | 846 | GL50803_17063 | 1008 | 0 | B |
| 1.2.4.1! | pyruvate dehydrogenase (acetyl-transferring) | 2632 | GL50803_3281 | 156 | 3.00E-39 | B |
| 4.1.1.1 | pyruvate decarboxylase | 75 | GL50803_9704 | 40.8 | 1.00E-05 | - |
| 2.3.1.12 | dihydrolipoyllysine-residue acetyltransferase | 1351 | GL50803_113021 | 647 | 0 | E, P |
| 6.2.1.1 | acetyl-CoA synthetase | 1718 | GL50803_13608 | 226 | 4.00E-60 | B |
| 6.2.1.13 | acetyl-CoA synthetase (ADP-forming) | 75 | GL50803_13608 | 507 | 5.00E-146 | A, B, P |
| 1.8.1.4! | dihydrolipoyl dehydrogenase | 2009 | GL50803_16125 | 450 | 2.00E-127 | - |
| 1.2.1.3 | aldehyde dehydrogenase (NAD^+^) | 1521 | GL50803_93358 | 70.1 | 4.00E-13 | - |
| 1.2.1.5 | aldehyde dehydrogenase [NAD(P)^+^] | 90 | GL50803_93358 | 65.5 | 6.00E-13 | - |
| 1.1.1.1 | alcohol dehydrogenase | 2659 | GL50803_93358 | 870 | 0 | B |
| 1.1.1.2 | alcohol dehydrogenase (NADP^+^) | 219 | GL50803_7260 | 240 | 6.00E-66 | E |
| 1.1.99.8 | [alcohol dehydrogenase (acceptor)](http://en.wikipedia.org/wiki/Alcohol_dehydrogenase_%28acceptor%29) | 102 | GL50803_3861 | 79.3 | 2.00E-17 | - |
| eutG | ethanol:NAD^+^ oxidoreductase | 62 | GL50803_93358 | 717 | 0 | B |
| 1.2.1.10 | acetaldehyde dehydrogenase | 611 | GL50803_93358 | 870 | 0 | B |

### 2. Enzymes from the TCA cycle in *Giardia*

| **EC** | **Enzyme name** | **Sequences in KEGG (#)** | **Best candidate** | **Bit-score** | **Evalue** | **Domains** |
| --- | --- | --- | --- | --- | --- | --- |
| 6.4.1.1 | pyruvate carboxylase | 526 | GL50803_113021 | 644 | 0 | P, B |
| 2.3.3.1 | citrate synthase | 1374 | GL50803_7195 | 359 | 2.00E-100 | B, A |
| 2.3.3.8 | ATP citrate (pro-S)-lyase | 166 | GL50803_13608 | 46.6 | 6.00E-07 | - |
| 4.1.3.6 | citrate lyase subunit alpha | 1028 | GL50803_38462 | 42.7 | 2.00E-04 | - |
| 4.2.1.3 | aconitate hydratase 1 | 1591 | GL50803_17063 | 89 | 2.00E-18 | - |
| 1.1.1.42 | isocitrate dehydrogenase | 1108 | GL50803_14785 | 43.9 | 1.00E-05 | - |
| 1.1.1.41 | isocitrate dehydrogenase (NAD^+^) | 508 | GL50803_11230 | 37.4 | 4.00E-04 | - |
| 1.2.4.2 | oxoglutarate dehydrogenase | 852 | GL50803_33769 | 52.8 | 3.00E-08 | - |
| 2.3.1.61 | 2-oxoglutarate dehydrogenase | 989 | GL50803_33769 | 51.2 | 5.00E-08 | - |
| 1.2.7.3 | 2-oxoglutarate synthase | 1528 | GL50803_22677 | 114 | 2.00E-27 | - |
| 6.2.1.4 | succinyl-CoA synthetase (GDP-forming) | 361 | GL50803_13608 | 48.9 | 1.00E-07 | - |
| 6.2.1.5 | succinyl-CoA synthetase (ADP-forming) | 2107 | GL50803_13608 | 435 | 2.00E-123 | B, A, E |
| 1.3.99.1 | succinate dehydrogenase | 4118 | GL50803_9089 | 56.6 | 5.00E-10 | - |
| 1.3.5.1 | succinate dehydrogenase (ubiquinone) | 1137 | GL50803_92246 | 62.8 | 6.00E-11 | - |
| 4.2.1.2 | fumarate hydratase | 1807 | GL50803_14259 | 84 | 5.00E-18 | - |
| 1.1.1.37† | malate dehydrogenase | 1349 | GL50803_3331 | 659 | 0 | B, E |

### 3. Enzymes from the oxidative phosphorylation in *Giardia*

| **EC** | **Enzyme name** | **Sequences in KEGG (#)** | **Best candidate** | **Bit-score** | **Evalue** | **Domains** |
| --- | --- | --- | --- | --- | --- | --- |
| 4.2.1.2 | fumarate hydratase | 1807 | GL50803_14259 | 84 | 5.00E-18 |  |
| **Complex I** | | | | | | |
| 1.6.5.3 | NADH dehydrogenase | 13702 | 12 proteins | 1080 | 0 | B, E, A |
| 1.6.99.3 | NADH dehydrogenase | 3956 | GL50803_33769 | 385 | 3.00E-108 | B, E, A |
| 1.6.99.5 | NADH dehydrogenase (quinone) | 3020 | GL50803_14058 | 83.6 | 1.00E-16 | - |
| **Complex II** | | | | | | |
| 1.3.99.1 | succinate dehydrogenase | 4118 | GL50803_39312 | 42.4 | 3.00E-04 | - |
| 1.3.5.1 | succinate dehydrogenase (ubiquinone) | 1137 | GL50803_9698 | 61.2 | 9.00E-12 | - |
| **Complex III** | | | | | | |
| 1.10.2.2 | ubiquinol-cytochrome c reductase | 1838 | GL50803_39312 | 42.4 | 6.00E-04 | - |
| **Complex IV** | | | | | | |
| 1.9.3.1 | cytochrome c oxidase | 4440 | GL50803_103783 | 38.1 | 0 | - |
| **ATP synthase** | | | | | | |
| 3.6.3.14 | F-type H^+^-transporting ATPase | 14587 | 21 proteins | 1800 | 0 | P, E |
| 3.6.3.10 | H^+^/K^+^-exchanging ATPase | 87 | GL50803_96670 | 2665 | 1.00E-170 | E |
| 3.6.3.6 | H^+^-transporting ATPase | 196 | 4 proteins | 590 | 0 | E |
| **Others** | | | | | | |
| 3.6.1.1 | inorganic pyrophosphatase | 1831 | 3 proteins | 1418 | 5.00E-05 |  |
| 2.7.4.1 | polyphosphate kinase | 703 | GL50803_8174 | 42.4 | 3.00E-04 | - |
